# Effect of Coconut and Chestnut Flour Supplementations on Texture, Nutritional and Sensory Properties of Baked Wheat Based Bread

**DOI:** 10.3390/molecules26154641

**Published:** 2021-07-30

**Authors:** Marianna Raczyk, Bartosz Kruszewski, Dorota Michałowska

**Affiliations:** 1 Department of Food Technology and Assessment, Institute of Food Sciences, Warsaw University of Life Sciences-SGGW, Nowoursynowska 159 C, 02-776 Warsaw, Poland; 2 Prof. Waclaw Dabrowski Institute of Agricultural and Food Biotechnology—State Research Institute, Rakowiecka 36, 02-532 Warsaw, Poland; dorota.michalowska@ibprs.pl

**Keywords:** coconut flour, chestnut flour, dietary fibre, protein, wheat bread, texture, sensory evaluation, functional food, PCA analysis

## Abstract

Wheat bread, produced by the single-phase method, is a common food consumed all over the world. Due to changes in lifestyle and nutritional trends, alternative raw materials are sought to increase the nutritional value and improve the taste of daily consumed products. Additionally, customers seek a wide variety of foods, especially when it comes to basic foods. Nuts, such as coconuts or chestnuts, might provide an attractive flavour with benefits to the nutritional quality. In this study, the effect of substituting wheat flour with coconut or chestnut flour (flour contribution level: 5, 10, 15, 30, 50% *w*/*w*), was evaluated in terms of the breads specific volume, texture, colour, nutritional composition, and dietary fibre fraction contents. Moreover, a sensory evaluation was conducted to assess potential consumer acceptance. Based on the consumer’s perception, the overall acceptance of bread with 15% *w*/*w* of coconut and chestnut flour was in privilege compared to the control sample. As a result, taking all of the tested parameters into account, the breads with 5, 10, and 15% supplementation of chestnut or coconut flour were still of good quality compared to the wheat bread and their fibre content was significantly higher.

## 1. Introduction

For most people in Europe bread is a basic and essential food product, which is consumed in everyday diet [1]. A great share of the bread available for sale is made of refined wheat flour. Those breads are often more attractive to consumers because of their soft crumbs, crispy crusts, light colour, and easy digestibility [2]. However, the nutritional value of such breads leaves much to be desired. In this context, bread enrichment with other cereal and non-cereal flours could have a positive effect on their nutritional value.

Chestnuts and coconuts are nuts cultivated in many regions of the world. They are globally popular for their flavour, and nutritional and health properties. European sweet chestnut (*Castanea sativa* Mill.) is mainly grown in continental European regions and has represented one of the most important and sustainable food resources of rural areas for many centuries. Chestnut flour (CH) is obtained by grinding dried chestnuts and it has a high starch content (50–60%), relatively high amount of sucrose (20–32%), high-quality proteins with essential amino acids (5–8%), dietary fibres (4–10%), and a low amount of lipids (2–4%) [2,3,4]. Predominant, free amino acids in chestnuts are aspartic acid, asparagine, and glutamic acid [5]. Crushed, uncooked chestnut kernels have a low glycemic index (GI = 54) [6]. Chestnut fruits are good sources of vitamins E, C, B1, B2, B3, and minerals Ca, P, K, Mg, S, Fe, Cu, Zn, and Mn [7]. Chestnuts are a good source of antioxidants and minerals, such as potassium and magnesium, which are linked to a reduction in the risk of cardiovascular diseases or stroke occurrence [8]. Sacchetti et al. [9] reported that chestnut flour is a good functional ingredient and may increase the content of some nutrients, having a positive effect on physical and nutritional properties in cereal-based foods. Chestnuts are often used as whole fruits, after various types of heat treatments, as an addition to meats, soups, creams, cakes, and puddings. Among popular products, there is also chestnut honey.

Coconut, as a fruit of palm trees (*Cocos nucifera* L.), needs a much warmer, tropical climate so it is mainly cultivated in Central and South America, Africa, and Asia. Coconuts in the form of whole fruits, as well as their parts and by-products obtained on daily basis by industries, is usually the main source of income for producing countries [10]. There is a large variety of products obtained from coconuts such as fruit, oil, water, milk, sugar, and coconut flour [3]. Coconut flour (CO) is a by-product of the coconut oil and milk production process. More precisely, it is the coconut pulp subjected to drying and grinding after the coconut oil/milk extraction process [11]. On the food market, it is more common to find defatted flour after oil extraction. The composition of coconut flour depends on which process it was previously subjected to, and what kind of extraction method was applied [12]. Coconut flour has a high content of protein, fat, and fibre in comparison to refined wheat flour [13,14]. Usually, it contains 3–5% of moisture, 4–6% of ash, 8–17% of fat, 15–22% of protein, 56–72% of total carbohydrates, and 10–56% dietary fibre. Additionally, it is a good source of K, Fe, Se, and Mg [11,12,15]. Coconut flour has five protein fractions, and albumin and globulin are predominant with a relatively high level of glutamic acid, aspartic acid, and arginine [10]. It contains eight essential amino acids and contributes to a well-balanced supply of proteins [16]. Due to the high nutritional value of coconut flour, in recent years numerous studies have demonstrated that it can be successfully used as a partial replacement of wheat or maize flour in the production of high-quality functional foods such as noodles, pasta, cookies, and energy bars [11,12,17,18]. Coconut flour has a relatively low glycemic index (GI) of 35, while wheat flour—approximately 85, hence it can be used by people with carbohydrate metabolism disorders [13]. It was shown that coconut flour supplementation can improve the GI and other health properties of many bakery products. Research by Trinidad et al. [19] shows a very strong negative correlation between the GI and dietary fibre content of the tested foods supplemented with coconut flour, as well as a significant reduction of total and LDL cholesterol, and triglycerides in blood serum.

In the food industry, there are ongoing studies on the production of cereal products with different additives, which could replace wheat flour. In the light of this trend, our research focuses on different substitution rates of coconut or chestnut flours to find the best proportions that benefit the nutritional and sensory properties of wheat bread.

## 2. Results and Discussion

### 2.1. Characteristics of the Flours

The characteristics of the flours, used as the main bread ingredients, are presented in Table 1. The water content of all flours applied in the study was within the recommended range [20]. It was noted that the water content of CH and CO flours was significantly lower than that of the wheat flour (Table 1). Flour moisture should not exceed 15% to keep the technological value safe enough to prevent the growth of undesirable microorganisms. The protein content of the analysed flours (g/100 g of dry matter) was the lowest in chestnut (6.93), then wheat (14.25), and the highest in coconut (19.47). The protein content of CH flour is relatively low, in agreement with the literature data [14,21]. A relatively high protein percentage of coconut flour in a range between 15 and 20 was also reported by other authors [10,14]. Even though the protein content of CO and CH flours is significant, those flours are gluten-free so it is difficult to create a characteristic bread structure, however, in combination with wheat flour, an acceptable texture can be achieved. Other studies also highlighted the potential of coconut utilization preceded by oil or milk production as it is rich in protein and fibre by-product [17]. Protein has a significant role in bodybuilding and may provide amino acids for body function [22]. Coconuts belong to oil plants, thus the coconut flour used in the study has a relatively high-fat content compared to wheat and chestnut (Table 1), as it was only partially defatted in the production. Among the analysed flours, chestnuts have the highest percentage of unsaturated fatty acids [2,7], but the fat of the coconut flour mostly consists of saturated fatty acids (approx. 90%) [10,11,19]. A notable difference in the share of ash among the tested flours was observed, in wheat four (0.71%), CH (1.98%), and CO (3.54%). CH flour is the richest in carbohydrates among studied flours (Table 1). The basic CH flour macronutrients evaluated in this study were not much different from the results reported by other authors [21,23]. On the other hand, CO flour composition differs depending on the study. Some research indicates much lower fat (10.9%) and protein (12.1%) contents [10,19], however, this is related to a different production technique, the kind of raw material, and intended application.

### 2.2. Dietary Fibre Evaluation

**Figure 1:**
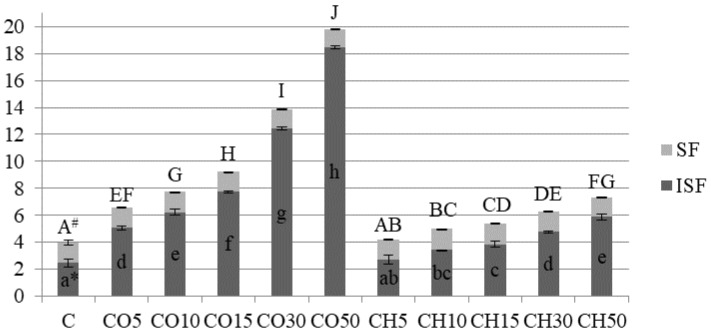
Fibre content in the analysed breads (g/100 g dry matter). The bars show the total fiber content divided into a dark grey part (insoluble fiber content, ISF) and a light grey part (soluble fiber content, SF). Bars represent the means ± standard deviations, (*n* = 4, for each bread type). * The values of ISF content, marked with different lowercase letters are significantly different (*p* < 0.05). # The values of SF content, marked with different capital letters are significantly different (*p* < 0.05).

**Figure 2:**
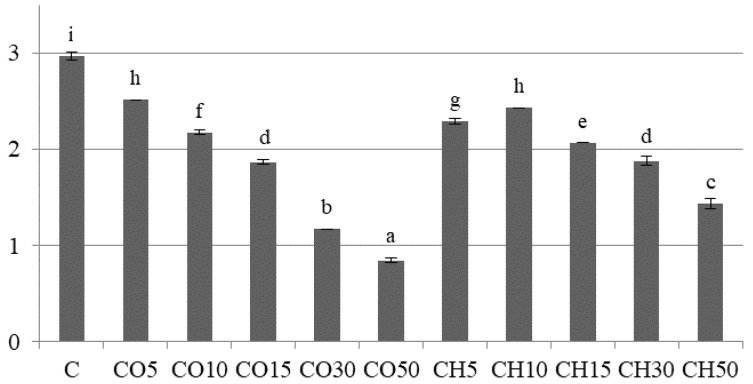
Specific volume of the analysed breads (mL/g). Bars represent means ± standard deviations, (*n* = 4, for each bread type). The values marked with different letters are significantly different (*p* < 0.05).

The total dietary fibre of coconut flour is diverse, and according to the literature amounts to 10–40% [11,17,19]. Our study shows that the share of total fibre in CO flour was 32.77 (g/100 g of dry matter) (Table 1). Fibre is known as a digestion-promoting factor and has a lot of positive effects on human health such as protective effects on diverticulum, constipation, colon cancer, obesity, diabetes, and cardiovascular diseases [24]. Coconuts and chestnuts are considered as one of the most fibre containing nuts. Among studied flours, coconut has the highest content of total dietary fibre. In the tested flours, the proportion of the insoluble fraction significantly prevails over a soluble fraction (Table 1). The significantly higher fibre content in coconut flour, compared to chestnut flour, had a negative impact on the breads volumes (Figure 1 and Figure 2). Based on the literature, dietary fibre increases the nutritional value of bread but usually at the same time alters the rheological properties of dough leading to a deterioration of the quality and sensorial properties of bread [25,26]. Other studies also reported that a major part of the total dietary fibre consists of an insoluble fraction [11,17,19]. In this research, CO, as well as CH flours had a significantly enlarged insoluble fibre content of prepared breads (Figure 1). Although the fibre has a positive nutritional effect, it can reduce the expansion of the gas cells leading to a lower volume of loaves and smaller porosity of the crumb.

### 2.3. Specific Volume, Colour and Texture of Breads

**Figure 3:**
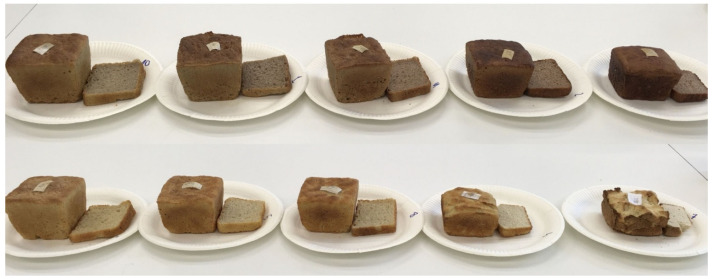
Crust and crumb appearance of the tested breads. The top row: bread supplemented with chestnut flour content from left to right 5, 10, 15, 30, and 50%. The bottom row: bread supplemented with coconut flour of content from left to right 5, 10, 15, 30, and 50%.

The volume of the loaves is generally affected when gluten-free flours substitute wheat flour [18,21]. The volume of breads with CO flour addition decreased proportionally to the increase in CO flour (Figure 2 and Figure 3, Figure S1A,B). The volume of breads with CH flour was also lower compared to the control (C) sample but decreased less drastically compared to the CO breads (Figure 2 and Figure 3). In the context of the loaves specific volume, the substitute with 5% CO and 10% CH flour can be used without a great lowering effect on this parameter (Figure 2). High protein, fibre, and fat content lead to a heavier dough which is difficult to “grow” in a fermentation step. Likewise, in some studies reported by other authors, an addition of chestnut and coconut flours decreased gluten-free breads volumes and caused a harder crumb [27]. A small replacement of wheat flour with chestnut flour (10–15%) can still lead to a good crumb structure, as was obtained in this and other studies [19,28].

The lightness (L*) of the crumb of the CH bread decreased proportionally to the addition of the CH flour. An opposite effect was noticed for the CO flour—the more flour share, the lighter the bread (Table 2). As chestnut flour has a relatively high sugar content, it may lead to a more compact structure, lower volume, and darker colour, caused by caramelization and the Millard reaction, as previously hypothesised by Demirkesen et al. [21]. The redness (a*) of loaves increased proportionally to the CH flour addition and decreased proportionally to the CO flour content compared to the C sample. The more CO flour in bread, the less yellowish colour compared to the C sample was measured (b*). This parameter slightly increased according to an increase in the CH flour in crumbs (Table 2).

**Figure 4:**
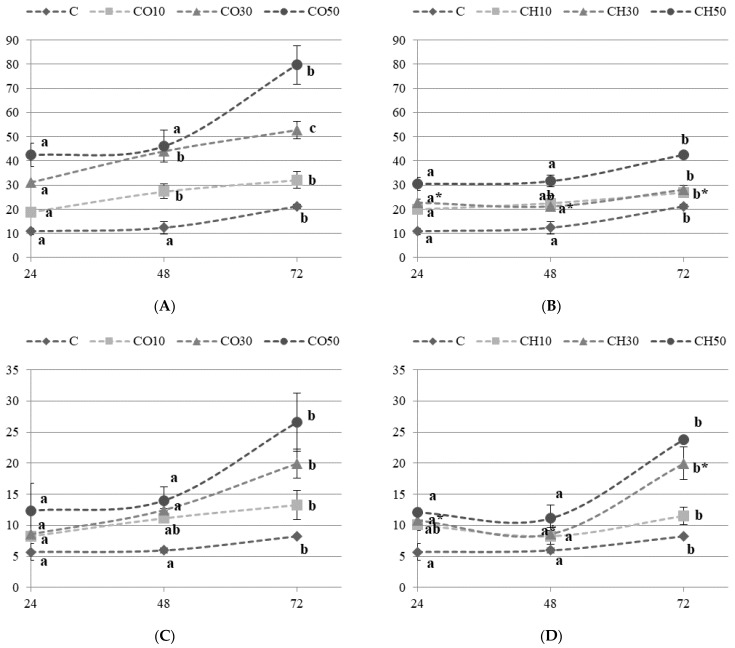
Crumb hardness [N] and gumminess during three days (24, 48 and 72 h) of storage for chosen experimental breads made with the formulation without or with coconut (CO) flour ((**A**)—hardness; (**C**)—gumminess) or chestnut (CH) flour ((**B**)—hardness; (**D**)—gumminess). Points represent the means ± standard deviations, (*n* = 6, for each bread type). The values within each type of bread, marked with different letters are significantly different (*p* < 0.05). * The homogeneous groups concern CH30 bread sample.

The hardness and gumminess of bread with 50% of CO and CH flour were the highest compared to other tested samples, and it increased significantly after 48 and 72 h of storage (Figure 4). A similar trend was observed in breads with 30% of CO and CH flours. Based on those results, we can already predict that such a texture would not be accepted by consumers. The increase in the fibre and sugar content is related to a harder bread crust, which was also proven in other studies [21,28,29]. On the other hand, the hardness and chewiness of breads with 10% of CO and CH flours were in line with the C sample, so it did not have a negative effect on the bread texture. Proportional to the CO or CH flour addition, lower resilience and cohesiveness of the crumb of breads after three days of storage at room temperature was observed (Appendix Table A1). Such results are in line with other studies [18,21,27]. The coarser crumb structure and reduced gas holding capacity of the doughs with the CO and CH flour is often attributed to the interference of the gluten network or the dough foam structure. Less gluten is formed and the breads with CO and CH are stiffer and less extensible.

### 2.4. Sensory Characteristics of Breads

**Figure 5:**
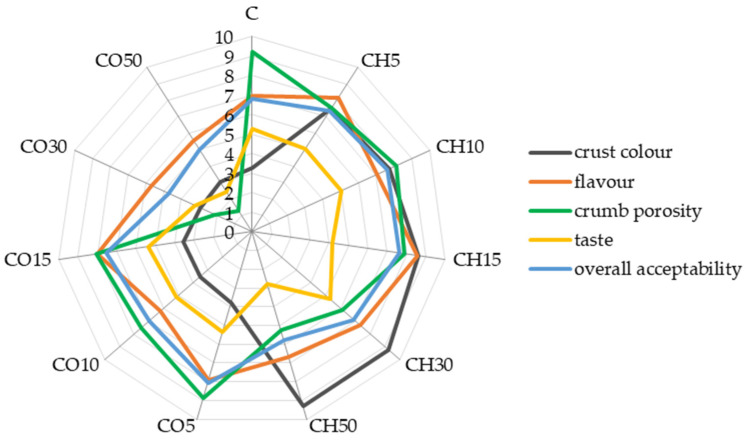
The scores of the sensory analysis of the obtained breads.

The results of the sensory evaluation indicate a significant effect of the bread supplementation with CH or CO flours on the sensory quality and overall consumer acceptability. A wheat flour replacement with 50% of CO or CH flours has a negative impact on all the parameters (Figure 5, Table S1) evaluated by the panel. Breads with 50% of CO or CH flours were very sweet, which would not be desired by consumers. The flavour of breads with 15% of CO and CH flours was significantly improved. All the variants with CO and CH flours had a negative effect on crumb porosity, however, 5, 10, and 15% of CO or CH flours had a positive effect on flavour and overall acceptability. Based on the results, it can be concluded that only breads with a CO and CH flour content of 5, 10, and 15% could be accepted by potential customers. Normally, consumers intend to buy healthy products but do not like to change their eating habits, therefore, only a small supplementation may not influence their acceptability. According to the panelists, a CH flour addition had a significant effect on the crust colour; it was more brownish compared to wheat bread. Although, the breads with CO flour were more yellowish in a sensory evaluation (Figure 5). This result is related to the colour of the raw material and a more intense browning process of the crust of bread with CH flour. Dark bread is much more often chosen by consumers due to the growing awareness of the presence of health-promoting compounds in whole flour, thus even if the colour is related to the different composition or browning process, the darker breads are still preferred by consumers [29,30]. Other studies reported that chestnut flour can be used in the production of cakes, breads, and biscuits to improve their sensory properties [17,21,27]. Other studies indicated that the chestnut flour containing breads showed a higher amount of volatiles compared to wheat bread [2], which could explain higher scores of flavour of breads supplemented with CH flour in this study.

### 2.5. The Comprehensive Overview of Obtained Breads Characteristics

**Figure 6:**
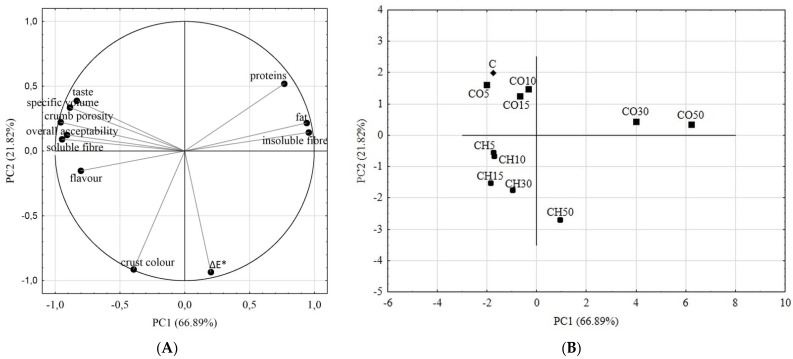
PCA analyses results. (**A**) Score plot, PC1 versus PC2 of all samples; (**B**) Score plot, PC1 versus PC2 of data from determinations used as variables.

In order to demonstrate comprehensively the alterations occurring in the types of the obtained breads, a chemometric analysis in the form of PCA was conducted on the qualified gathered data. In the PCA analysis, ten principal components were created, of which the first two explained 88.71% of the total variability. Figure 6A shows the distribution of samples in two-dimensional space relative to the first and second principal components. The attributes that most differentiated the samples of breads with CO flour were the content of protein, fat, and insoluble fibre (Figure 6B). The bread CO5, with a 5% replacement of wheat flour with CO flour, was the most similar to the control sample in terms of the changes studied. The slight addition of CO flour proved beneficial in terms of nutritional value—insoluble fibre content doubled, and fat content increased by half compared to wheat bread. This implied positive changes in the overall sensory quality of the bread with a slight decrease in volume. The CO5 loaves had better flavour, crust colour, and received the best score of all samples in terms of overall acceptability. Greater changes in wheat bread characteristics were noticed when 10 and 15% of wheat flour were substituted with CO flour. This resulted in a further increase in insoluble fibre and fat content by approximately 3 times in both samples (CO10 and CO15). Changes in the amounts of these substances significantly affected the texture quality of the breads. Also, similar reductions in the loaves volume and deterioration in crumb porosity were noted for those samples. However, taste, flavour, and overall acceptability were similar or higher compared to wheat bread (C). The PCA analysis generated another group of samples of breads with 30 and 50% of CO flour (CO30 and CO50) which were of the highest content of insoluble fibre and fat among studied samples. An increase in the protein, fibre, and fat content had a significantly negative effect on the texture of the loaves and its overall appearance, including taste, flavour, irregular colour of the crust, and low volume. In general, replacing wheat flour with 5, 10, and 15% of CO flour might be beneficial in terms of both nutritional and sensory values.

Due to the different chemical compositions, the effect of CH flour on the physicochemical and sensory characteristics of wheat bread was clearly different from that of the CO flour (Figure 6A). The attributes that mostly differentiated the breads with CH flour were the flavour, crust colour, and total colour difference ΔE* of crumb (Figure 6B). Substitution of 5 and 10% wheat flour (CH5 and CH10) resulted in a slight increase in insoluble fibre content and a slight decrease in protein content. Specific volume decreased by approximately 23%, but both breads were distinguished by high scores of the sensory evaluation. The presence of CH flour significantly affected the bread colour, creating a darker brown crust and crumb as it was determined by the panelists and instrumental assessment. A further increase in the proportion of CH flour up to 15, 30, and 50% resulted in an increase in insoluble fibre, fat, and a decrease in protein content. Moreover, bread with 50% of chestnut flour (CH50) was evaluated as very sweet. Also, the specific volume of the loaves dropped to a low level. All the above characteristics led the PCA analysis to classify CH50 bread separately. Among breads with CH flour, it is difficult to clearly define which content of the flour, 15% or 30%, is still beneficial to the overall quality, and this needs further research. Certainly, a 50% of this flour is not advisable in a bread-making technology.

## 3. Materials and Methods

### 3.1. Chemicals

All of the reagents and solvents used, of the reagent grade purity, they were obtained from Avantor Performance Materials Poland S.A. (Gliwice, Poland).

### 3.2. Preparation of Breads

The breads were made on a laboratory scale, and doughs were obtained by the single-phase method. The following ingredients were used to prepare it: wheat (country of origin: Teresin, Poland) flour type 750 (mineral content 0.70–0.78% of dry matter), chestnut (*Castanea sativa* Mill., country of origin: Emilia-Romagna, Italy) flour, coconut (*Cocos nucifera* L., country of origin: Sri Lanka) flour, dried baker’s yeast (*Saccharomyces cerevisiae* L.), salt and water were added to obtain a dough of a yield of 165% (Table 3). All ingredients were mixed in an SP-800A SPAR mixer (Food Machinery, Taiwan) for 5 min in a stainless-steel bowl. The doughs were fermented in a proofer chamber at 30 °C and 70% humidity for 1.5 h, kneaded after 1 h. Then 250 g of dough were weighed into the forms and rested for 1 h in the proofer chamber (in the same conditions as above). The doughs were baked in the Sveba Dahlen oven (Fristad, Sweden) at 220 °C for 30 min. After baking, all bread loaves were cooled at room temperature. Then, samples were packed in a plastic box and kept in dark at 25 °C and 60% RH for further analysis. Products of two independent batches were analysed.

### 3.3. Specific Volume of Breads

The mass of the loaves was determined using a digital weight with 0.01 g accuracy. The loaf volume was evaluated using a standard rapeseed displacement method. Bread-specific volume was determined according to the AACC Approved Method 10-05.01 [31] and expressed as the volume/weight ratio of baked bread (mL/g). 

### 3.4. Moisture and Ash Determination

The flour and bread crumb moisture was determined by the drying method according to the AACC standard method 44-15.02 [32]. 5 g of each sample was weighed on an analytical balance into metal dishes and then dried in a SUP-65 W laboratory dryer (Wamed, Poland) at 130 °C for 60 min. After drying, the dishes were placed in a desiccator to cool and then reweighed. The moisture was calculated from the mass difference. The ash content was determined based on the AACC standard method 08-01.01 [33]. 5 g of each flour was weighted into an ash dish. Then samples were placed in a muffle furnace at 900 °C for 1 h. After cooling, the samples were weighed, and the ash contents were calculated.

### 3.5. Determination of Proximal Nutritional and Energy Values

Protein content was determined according to the Kjeldahl method [34] (correction factor of 5.7), whereas fat was extracted and determined by the Soxhlet apparatus using diethyl ether as solvent. Total dietary fibre content, including insoluble and soluble fractions, was determinate by the enzymatic–gravimetric method according to AOAC 991.43, and AACC 32-07 procedures [35,36]. For these determinations, Fibertec^TM^ E System (FOSS, Hilleroed, Denmark) was used. The carbohydrates content was calculated by subtracting the values of the protein, fat, moisture, total dietary fibre, and ash content from 100. The energy values (kcal) were calculated using conversion factors according to EU Regulation No 1169/2011 (4 kcal per g for protein and carbohydrates, 9 kcal per g for fat, 2 kcal per g for total fibre). The conversion factor for kilojoules is 1 kcal = 4.184 kJ.

### 3.6. Colour Determination of Breads

The crumb colour of baked loaves was evaluated using a colorimeter Konica Minolta CM-3600d (Tokyo, Japan), and calibrated against black and white plate standards. Crumb colour was determined at the middle point of the central 2 cm thick slice. The parameters of the device in the reflection mode were set as follows: a standard observer of 10°, and an illuminate D65. The measurements were performed at room temperature through a diaphragm of 3 cm diameter. The colour was expressed in the CIE L*a*b* scale, and the parameters determined were: L* (lightness), a* (redness), and b* (yellowness).

### 3.7. Evaluation of Breads Texture

The crumb texture was evaluated applying texture profile analysis (TPA) (Stable Micro Systems Ltd., Godalming, UK). TPA test was carried out using a cylindrical mandrel of a diameter of 25 mm, which penetrated the crumb to a depth of 9 mm with a crosshead speed of 40 mm/min. Measurements were carried out on middle slices of 20 mm thickness taken from the centre of each kind of experimental loaves (in triplicate). The bread samples were packed in a plastic box and kept in dark at room temperature (at 25 °C and 60% RH). Measurements were taken 24, 48, and 72 h after baking. Each slice was compressed twice to give texture profile charts obtained in the Texture Expert Exceed software, from which following textural parameters were determined: hardness (peak force of the first compression cycle, N), gumminess (ratio of the time duration of force input during the second compression to that during the first compression, dimensionless), resilience (area during the withdrawal of the penetration, divided by the area of the first penetration, dimensionless), cohesiveness (ratio of positive force area during the second compression to that during the first compression area, dimensionless), and chewiness (hardness_cohesiveness_gumminess, in N) [37].

### 3.8. Sensory Evaluation of Breads

Sensory evaluation of breads was carried out by ten trained panelists on the day of production (2 h after baking). The questionnaire included a 10-point scaling method based on the Polish norm (PN-A-74108, 1996). The same group of ten panelists received samples of coded bread in two sessions—at the rate of 6 per session (control and coconut or chestnut supplemented samples). During the testing sessions, panelists had access to drinking water to clean the palate between evaluations. Panelists were trained using ISO 8586-1:2012, ISO 8586-2:2014, and ISO 11036:2020. The following characteristics were assessed: crust colour (0 = yellowish and 10 = brownish), taste (0 = sweet and 10 = bitter), flavour (0 = unwanted and 10 = aromatic, savoury), crumb porosity (0 = uneven, compacted and 10 = even), and an overall acceptability (0 = dislike very much and 10 = like very much).

### 3.9. Calculations and Statistics

All data in tables were presented as the mean with standard deviations. Unless otherwise stated, determinations were performed in triplicate. The statistical program Statistica 13.3 (TIBCO Software Inc., San Francisco, CA, USA) was used to develop the results. The effect of the type of flour, as well as the effect of the wheat flour replacement with coconut or chestnut flours on nutritious composition and/or physicochemical properties was analysed using the one-way analysis of variance (ANOVA). To evaluate the differences between average values for data that were normally distributed, the Tukey HSD test was used with a significance level of α = 0.05. If the tested data did not come from a normal distribution, the Kruskal–Wallis test was used instead.

The gathered quantitative data were used in the chemometric analysis in principal component analysis (PCA) in order to show the affinity of the obtained wheat-coconut or wheat-chestnut bread loaves towards wheat bread treated as a control sample. The results of the determinations performed were qualified for PCA analysis based on a correlation score with the first or second principal component at a level of at least 0.7. According to the generated factor loadings matrix, data from the following analyses of breads were qualified for PCA classification: insoluble and soluble fibre, protein and fat content, specific volume, total colour difference ΔE*of crumb, and sensory characteristics (crust colour, flavour, crumb porosity, taste, and overall acceptability).

## 4. Conclusions

Among the analysed flours, the lowest moisture, carbohydrates, soluble dietary fibre content, and the highest ash, protein, fat, and insoluble dietary fibre content were specified in coconut flour. The insoluble dietary fibre fraction content of prepared breads was significantly increased by replacing part of the wheat flour with CH or CO flour. The volumes of breads with CO or CH flour supplementation decreased proportionally to the flour content, compared to wheat bread. A sensory evaluation points out that breads with 30 and 50% of CO and CH flour did not have a positive effect on the tested parameters, however, the supplementation with 5, 10, and 15% improved a breads taste, flavour, and overall acceptability. As a result, taking all the tested parameters into account, the breads with 5, 10, and 15% supplementation of CH or CO flours were still of good quality compared to the wheat bread, and their fibre content was significantly higher. Therefore, chestnut flour, as well as coconut flour, can be great ingredients for making nutritionally enhanced foods as well as functional foods.

## Figures and Tables

**Table 1:** Proximate macronutrients content (g/100 g of dry matter) and energy value of the flours.

	Wheat Flour	Coconut (CO) Flour	Chestnut (CH) Flour
Moisture (%)	12.75 ± 0.01 ^c^	4.37 ± 0.01 ^a^	6.80 ± 0.01 ^b^
Protein	14.25 ± 0.04 ^b^	19.47 ± 0.12 ^c^	6.93 ± 0.18 ^a^
Fat	2.03 ± 0.01 ^a^	24.83 ± 0.16 ^b^	3.78 ± 0.02 ^a^
Ash	0.71 ± 0.01 ^a^	3.54 ± 0.01 ^c^	1.98 ± 0.01 ^b^
Carbohydrates	67.41 ± 0.02 ^b^	15.02 ± 0.08 ^a^	68.02 ± 0.06 ^b^
Insoluble dietary fibre	1.75 ± 0.04 ^a^	31.70 ± 0.11 ^c^	10.91 ± 0.68 ^b^
Soluble dietary fibre	1.10 ± 0.25 ^a^	1.07 ± 0.08 ^a^	1.58 ± 0.13 ^b^
Total dietary fibre	2.85 ± 0.22 ^a^	32.77 ± 0.19 ^c^	12.49 ± 0.81 ^b^
Energy value (kJ)	1489.6 ± 35.7 ^a^	1785.1 ± 9.1 ^b^	1500.9 ± 13.9 ^a^
Energy value (kcal)	356.0 ± 8.5 ^a^	426.7 ± 2.2 ^b^	358.7 ± 3.3 ^a^

The values represent the mean ± standard deviation (*n* = 3, for each flour type). The values in a row with different letters are significantly different (*p* < 0.05).

**Table 2:** Colorimetric parameters and proximate macronutrients composition of the analysed breads.

.	C	CO5	CO10	CO15	CO30	CO50	CH5	CH10	CH15	CH30	CH50
Colorimetric parameters of crumb ‡											
L*	66.89 ± 0.31 ^d^	67.91 ± 0.27 ^e^	68.92 ± 0.60 ^f^	70.55 ± 0.44 ^g^	75.43 ± 0.24 ^h^	79.01 ± 0.80 ^i^	60.77 ± 0.25 ^c^	54.64 ± 0.35 ^b^	54.07 ± 0.31 ^b^	51.84 ± 0.37 ^a^	52.18 ± 0.32 ^a^
a*	3.06 ± 0.08 ^d^	3.03 ± 0.07 ^cd^	3.00 ± 0.12 ^cd^	2.84 ± 0.09 ^c^	2.36 ± 0.04 ^b^	1.99 ± 0.13 ^a^	4.26 ± 0.03 ^e^	5.45 ± 0.07 ^f^	6.20 ± 0.06 ^g^	8.44 ± 0.05 ^h^	8.55 ± 0.22 ^h^
b*	21.91 ± 0.27 ^g^	22.23 ± 0.17 ^gh^	22.54 ± 0.22 ^h^	21.86 ± 0.22 ^g^	19.81 ± 0.35 ^f^	17.48 ± 0.35 ^e^	13.30 ± 0.20 ^a^	13.66 ± 0.16 ^ab^	14.16 ± 0.14 ^b^	15.65 ± 0.17 ^c^	16.48 ± 0.49 ^d^
ΔE*	-	1.08 ± 0.24 ^a^	2.15 ± 0.54 ^b^	3.67 ± 0.45 ^c^	8.83 ± 0.23 ^d^	12.95 ± 0.80 ^f^	10.63 ± 0.26 ^e^	14.96 ± 0.25 ^g^	15.31 ± 0.24 ^g^	17.17 ± 0.31 ^h^	16.63 ± 0.30 ^h^
Macronutrients composition (g/100 g dry matter) of breads#											
moisture (%)	35.39 ± 0.01 ^e^	34.04 ± 0.01 ^a^	35.41 ± 0.02 ^e^	35.62 ± 0.01 ^f^	35.96 ± 0,02 ^h^	35.43 ± 0.01 ^e^	35.23 ± 0,02 ^c^	35.31 ± 0.02 ^d^	35.61 ± 0.01 ^f^	35.70 ± 0.01 ^g^	34.69 ± 0.02 ^b^
proteins	13.43 ± 0.25 ^d^	13.02 ± 0.35 ^cd^	13.19 ± 0.36 ^d^	13.29 ± 0.21 ^d^	15.53 ± 0.13 ^e^	17.79 ± 0.07 ^f^	12.41 ± 0.69 ^cd^	12.87 ± 0.32 ^cd^	11.87 ± 0.13 ^bc^	11.37 ± 0.08 ^ab^	10.52 ± 0.18 ^a^
fat	1.83 ± 0.02 ^a^	2.83 ± 0.04 ^f^	3.99 ± 0.04 ^g^	5.12 ± 0.04 ^h^	8.69 ± 0.03 ^i^	14.33 ± 0.06 ^j^	1.90 ± 0.02 ^ab^	1.98 ± 0.04 ^bc^	2.06 ± 0.04 ^c^	2.32 ± 0.04 ^d^	2.63 ± 0.04 ^e^
ash	1.42 ± 0.03 ^a^	1.72 ± 0.04 ^c^	2.08 ± 0.03 ^e^	2.41 ± 0.05 ^f^	3.42 ± 0.03 ^h^	4.70 ± 0.05 ^i^	1.56 ± 0.04 ^b^	1.71 ± 0.05 ^c^	1.86 ± 0.04 ^d^	2.30 ± 0.06 ^f^	2.86 ± 0.03 ^g^
carbohydrates	44.68 ± 0.73 ^fg^	41.78 ± 0.50 ^e^	37.58 ± 0.69 ^d^	34.31 ± 0.21 ^c^	22.50 ± 0.07 ^b^	7.91 ± 0.06 ^a^	44.68 ± 1.04 ^g^	43.15 ± 0.29 ^fg^	43.18 ± 0.38 ^fg^	42.02 ± 0.19 ^ef^	41.99 ± 0.06 ^e^
energy (kcal)	253.9 ± 0.9 ^d^	257.8 ± 0.5 ^e^	254.4 ± 0.4 ^d^	254.9 ± 0.2 ^d^	258.1 ± 0.3 ^e^	271.4 ± 0.3 ^f^	253.8 ± 0.5 ^d^	251.8 ± 0.3 ^c^	249.5 ± 0.2 ^b^	246.9 ± 0.5 ^a^	248.2 ± 0.2 ^ab^
energy (kJ)	1062.2 ± 3.6 ^d^	1078.6 ± 1.0 ^e^	1064.6 ± 1.7 ^d^	1066.5 ± 0.7 ^d^	1079.9 ± 1.1 ^e^	1135.5 ± 1.2 ^f^	1061.9 ± 2.1 ^d^	1053.3 ± 1.1 ^c^	1043.9 ± 0.8 ^b^	1033.2 ± 2.2 ^a^	1038.6 ± 0.7 ^ab^

‡ *n* = 12, for each bread type. # *n* = 6, for each bread type and ‡ *n* = 6, for each crumb of a bread type. The values represent the means ± standard deviations. The values in a row with different letters are significantly different (*p* < 0.05).

**Table 3:** Recipe ingredients of studied breads [% content].

Ingredient	C *	CO5	CO10	CO15	CO30	CO50	CH5	CH10	CH15	CH30	CH50
Wheat flour	59.7	56.8	53.8	50.8	41.8	29.9	56.8	53.8	50.8	41.8	29.9
Water	38.8	38.8	38.8	38.8	38.8	38.8	38.8	38.8	38.8	38.8	38.8
Salt	0.9	0.9	0.9	0.9	0.9	0.9	0.9	0.9	0.9	0.9	0.9
Yeast	0.5	0.5	0.5	0.5	0.5	0.5	0.5	0.5	0.5	0.5	0.5
Coconut flour	-	3.0	6.0	9.0	17.9	29.9	-	-	-	-	-
Chestnut flour	-	-	-	-	-	-	3.0	6.0	9.0	17.9	29.9

* C: control (wheat bread), CO: bread with coconut flour addition, CH: bread with chestnut flour addition; 5, 10, 15, 30, 50: percentage replacement of wheat flour with coconut/chestnut flours.

## Data Availability

The data presented in this study are available in Supplementary Material.

## Supplementary Materials

The following are available online, Figure S1: The appearance of tested breads compared with wheat bread as control sample. (A) from left to right: control bread and breads supplemented with chestnut flour content 10, 30 and 50%; (B) from left to right: control bread and breads supplemented with coconut flour content 10, 30 and 50%, Table S1: The scores of the sensory analysis of the obtained breads.

## Sample Availability

Samples of the compounds are not available from the authors.

## Abbreviations

CO—coconut flour; CH—chestnut flour; C—control sample.

## Appendix A

**Table A1:** Crumb texture parameters for the selected analysed breads after 24, 48 and 72 h of storage at room temperature.

	Resilience	Cohesiveness	Chewiness (N)	Resilience	Cohesiveness	Chewiness (N)
	C			CO10		
24 h	0.89 ± 0.03 ^a^	0.53 ± 0.01 ^b^	5.05 ± 0.38 ^a^	0.84 ± 0.07 ^a^	0.44 ± 0.01 ^a^	6.87 ± 0.54 ^a^
48 h	0.90 ± 0.05 ^a^	0.43 ± 0.05 ^a^	4.75 ± 1.25 ^a^	0.93 ± 0.11 ^a^	0.41 ± 0.04 ^a^	10.36 ± 1.18 ^b^
72 h	0.87 ± 0.01 ^a^	0.39 ± 0.01 ^a^	7.28 ± 0.12 ^b^	0.89 ± 0.08 ^a^	0.41 ± 0.03 ^a^	11.70 ± 1.03 ^b^
	CO30			CO50		
24 h	0.87 ± 0.12 ^b^	0.41 ± 0.02 ^b^	7.43 ± 0.68 ^a^	0.48 ± 0.03 ^a^	0.29 ± 0.07 ^a^	6.32 ± 2.00 ^a^
48 h	0.57 ± 0.02 ^a^	0.28 ± 0.03 ^a^	7.07 ± 1.05 ^a^	0.50 ± 0.05 ^a^	0.26 ± 0.02 ^a^	7.03 ± 0.74 ^a^
72 h	0.67 ± 0.10 ^ab^	0.38 ± 0.04 ^b^	13.96 ± 1.29 ^b^	0.48 ± 0.09 ^a^	0.33 ± 0.03 ^a^	12.67 ± 1.96 ^b^
	CH10			CH30		
24 h	0.93 ± 0.10 ^a^	0.45 ± 0.01 ^a^	9.32 ± 1.08 ^a^	0.67 ± 0.10 ^a^	0.38 ± 0.04 ^a^	7.43 ± 0.6 8 ^a^
48 h	0.91 ± 0.03 ^a^	0.42 ± 0.03 ^a^	7.58 ± 1.38 ^a^	0.87 ± 0.12 ^a^	0.41 ± 0.02 ^a^	9.70 ± 0.75 ^b^
72 h	0.90 ± 0.07 ^a^	0.44 ± 0.01 ^a^	10.42 ± 1.65 ^a^	0.87 ± 0.09 ^a^	0.38 ± 0.03 ^a^	14.29 ± 0.85 ^c^
	CH50					
24 h	0.94 ± 0.06 ^a^	0.40 ± 0.02 ^b^	11.36 ± 1.05 ^a^			
48 h	0.97 ± 0.05 ^a^	0.35 ± 0.03 ^b^	12.34 ± 0.15 ^b^			
72 h	0.80 ± 0.14 ^a^	0.18 ± 0.01 ^a^	16.04 ± 0.76 ^c^			

Values represent means ± standard deviation, (*n* = 6, for each bread type). Respectively for each bread the values in a column with different letters are significantly different (*p* < 0.05).
