# Supplementary material for: Effect of Coconut and Chestnut Flour Supplementations on Texture, Nutritional and Sensory Properties of Baked Wheat Based Bread
Source: Molecules. 2021 Jul 30;26(15):4641. doi: 10.3390/molecules26154641 (PMC8347958; doi:10.3390/molecules26154641)
Supplement: Supplementary file 1 [file molecules-26-04641-s001.zip › molecules-1303297-supplementary.pdf]

Effect of coconut and chestnut flour supplementations on texture, nutritional and sensory properties of baked wheat based bread

Marianna Raczyk [1,*], Bartosz Kruszewski [1,*] and Dorota Michałowska [2]

[1] Department of Food Technology and Assessment, Institute of Food Sciences, Warsaw University of Life Sciences – SGGW, Nowoursynowska 159 C, Warsaw, 02-776, Poland
[2] Prof. Waclaw Dabrowski Institute of Agricultural and Food Biotechnology – State Research Institute, Rakowiecka 36, Warsaw, 02-532, Poland; dorota.michalowska@ibprs.pl
* Correspondence: marianna_raczyk@sggw.edu.pl (MR); Tel.: +48-22-593-75-40, bartosz_kruszewski@sggw.edu.pl (BK); Tel.: +48-22-593-75-20

Supplementary material

1. Experimental breads

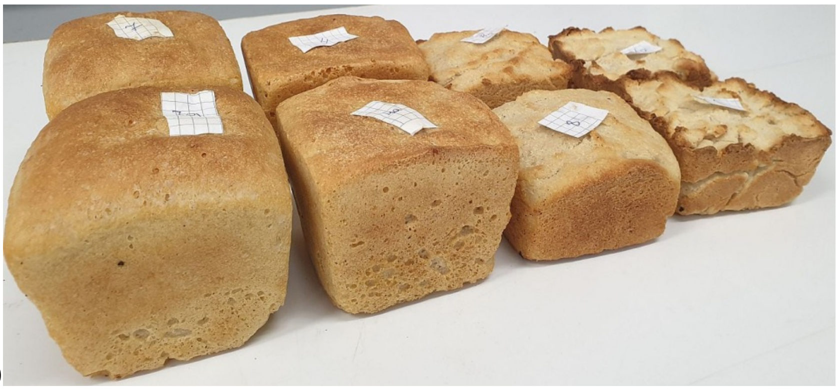

A)

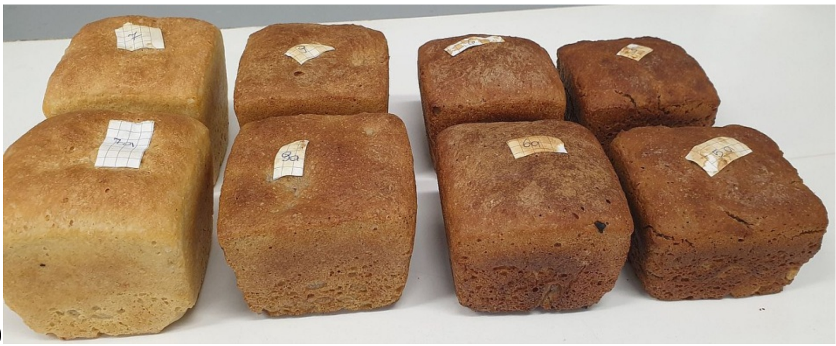

B)

Figure S1. The appearance of tested breads compared with wheat bread as control sample. **A)** from left to right: control bread and breads supplemented with chestnut flour content 10, 30 and 50%; **B)** from left to right: control bread and breads supplemented with coconut flour content 10, 30 and 50%.

2. Sensory evaluation scores

Table S1. The scores of the sensory analysis of the obtained breads.

	crust color	flavour	crumb porosity	taste	overall acceptability
**C**	3.3 ± 0.7 ^{a}	6.9 ± 0.3 ^{ab}	9.2 ± 0.3 ^{e}	5.3 ± 0.4 ^{c}	6.8 ± 0.4 ^{b}
**CO5**	3.8 ± 0.6 ^{a}	7.9 ± 0.3 ^{b}	8.9 ± 0.1 ^{e}	5.4 ± 0.3 ^{c}	8.1 ± 0.3 ^{c}
**CO10**	3.5 ± 0.9 ^{a}	6.2 ± 0.6 ^{a}	7.5 ± 0.4 ^{d}	5.1 ± 0.5 ^{c}	6.9 ± 0.3 ^{b}
**CO15**	3.6 ± 0.8 ^{a}	8.0 ± 0.5 ^{b}	8.1 ± 0.5 ^{d}	5.4 ± 0.5 ^{c}	7.6 ± 0.5 ^{bc}
**CO30**	2.9 ± 0.5 ^{a}	5.7 ± 0.4 ^{a}	2.1 ± 0.2 ^{b}	3.3 ± 0.2 ^{ab}	4.7 ± 0.2 ^{a}
**CO50**	3.0 ± 0.4 ^{a}	5.6 ± 0.3 ^{a}	1.3 ± 0.2 ^{a}	2.4 ± 0.2 ^{a}	5.0 ± 0.4 ^{a}
**CH5**	7.5 ± 0.5 ^{b}	8.2 ± 0.7 ^{bc}	7.6 ± 0.5 ^{d}	5.0 ± 0.4 ^{c}	7.3 ± 0.2 ^{bc}
**CH10**	7.8 ± 0.5 ^{b}	7.0 ± 0.5 ^{b}	8.1 ± 0.2 ^{d}	5.0 ± 0.3 ^{c}	7.6 ± 0.3 ^{bc}
**CH15**	8.6 ± 0.8 ^{b}	8.6 ± 0,7 ^{c}	7.9 ± 0.3 ^{d}	4.1 ± 0.3 ^{b}	7.6 ± 0.4 ^{bc}
**CH30**	9.2 ± 0.6 ^{b}	7.3 ± 0.3 ^{b}	6.1 ± 0.4 ^{c}	5.3 ± 0.5 ^{c}	6.9 ± 0.3 ^{b}
**CH50**	9.3 ± 0.5 ^{b}	6.7 ± 0.5 ^{ab}	5.3 ± 0.2 ^{c}	2.8 ± 0.2 ^{a}	5.8 ± 0.4 ^{ab}

n = 10, for each bread type. The values represent means ± standard deviation. The values in a column with different letters are significantly different ($p < 0.05$).
